# Dysfunction of peroxisomes as one of the possible causes of multiple organ dysfunction syndrome development

**DOI:** 10.1186/cc13421

**Published:** 2014-03-17

**Authors:** HK Osipenko, B Marochkov

**Affiliations:** 1 A. Kuleshov Mogilev State University, Mogilev, Belarus; 2 Mogilev Regional Hospital, Mogilev, Belarus

## Introduction

This study demonstrates that low level of plasmalogens (Pls) is an important marker of peroxisomal dysfunction. The primary- OH group in glycerol Pls was not substituted by the acyl group (fatty acid) as in diacylphospholipids but by the aldehydogenic alkenyl group (fatty aldehyde) found in the form of vinyl ether [1]. It is known that there is disruption of several organ systems in diseases connected with peroxisomal dysfunction, as in the case of multiple organ failure.

## Methods

The objects of study were 18 people with multiple organ failure (35.6 ± 8.7 years) of various etiologies. The blood of 16 healthy volunteers aged 37.7 ± 3.2 years served as control. The preanalytical phase was to prepare a solution of ethyl esters of fatty acids (FAc) and diethylacetals of fatty aldehydes (FAl) of blood plasma samples by acidic ethanolysis. Analysis of FAl and FAc in plasma was carried out using capillary gas-liquid chromatography. Quantitative evaluation of the analytes was performed as a mass percentage of the FA and FAl amount. Statistical analysis was performed using the Mann-Whitney U test (*P *< 0.05).

## Results

In contradistinction to triglycerides, diacylphospholipids and Pls participate in exchange of polyunsaturated fatty acids (PUFA) with a large number of double bonds, such as primarily arachidonic and docosahexaenoic PUFA, acting as an intermediate station, through which these fatty acids are transported to cell membranes [1]. Thus, the ratio of level of FAl to the level of aforementioned blood plasma PUFA may reflect a change in the share of Pls relative to diacylphospholipids. According to our data, at MODS the ratio of fatty aldehydes to amount of arachidonic and docosahexaenoic PUFA is 0.16, while in the control group the figure is 0.27 (*P *< 0.001). Thus, we can conclude that the level of Pls towards diacylphospholipid plasma levels of the experimental group decreased by approximately 40%. Currently, there is evidence that there is a reduction of cholesterol levels in blood plasma of patients with the diseases associated with peroxisome biogenesis disorders[2]. In our study, patients had low levels of plasma total cholesterol (124.6 ± 28.7 mg/dl), which may also indicate peroxisome dysfunction in MODS patients.

## Conclusion

On the basis of determined deficiency of analyzed compound levels, we concluded the possibility of peroxysomal dysfunction in MODS.
